# Editorial: Meaning in everyday life: working, playing, consuming, and more

**DOI:** 10.3389/fpsyg.2023.1221799

**Published:** 2023-06-20

**Authors:** Pninit Russo-Netzer, Joshua Hicks

**Affiliations:** ^1^School of Advanced Studies, Achva Academic College, Arugot, Israel; ^2^Department of Psychological and Brain Sciences, Texas A&M University, College Station, TX, United States

**Keywords:** meaning in life, wellbeing, growth, meaningful life, experiences, positive psychology, existential psychology

The search for meaning lies at the very core of human psychology, from young children asking “why” questions in an effort to make sense of the world to adults seeking more meaning through work, hobbies, and the marketplace. While scholarly interest in meaning dates back centuries, researchers have only gradually begun to explore the search for meaning in a systematic manner. Existing empirical research has begun uncovering many psychological, spiritual, and physical effects of meaning and purpose for individuals, organizations, and communities, but many gaps remain. The current collection addresses one particular gap: how people seek, find, and create meaning through everyday activities. It includes 12 articles that highlight the latest innovative work to advance new knowledge about meaning in life by understanding how people pursue, find, and experience meaning through everyday activities.

## “Connecting the dots”: past, present, and future in the science of meaning in life

The desire for a meaningful life is one of the cornerstones of human existence. Defined as “shared mental representations of possible relationships among things, events, and relationships” (Baumeister, 1991, p. 15), the way in which people perceive and prioritize meaning in their lives has changed over time in response to historical and cultural shifts. While the study of meaning is centuries old in philosophy, it is relatively new in fields that focus on how to enhance and enrich human functioning (e.g., Russo-Netzer and Vos, in press). Even though scholars have begun to develop sophisticated ways to examine how people search for and create meaning in their lives, the study of meaning has been relatively circumscribed to understanding the concept of meaning and how it contributes to wellbeing and coping (e.g., Janoff-Bulman and Yopyk, 2004; Steger et al., 2009; Linley and Joseph, 2011; Steger, 2012; Czekierda et al., 2017; Russo-Netzer, 2018). Several studies in this collection further support and extend the importance of meaning in life as a resource for promoting wellbeing, whether it is in the context of post-traumatic growth or life satisfaction. Kalashnikova et al. compare suicidal and non-suicidal patients' views on the meaning of life as a resource for coping with psychological crises. The study highlights the importance of meaning in life as a coping resource for psychological distress and suggests that enhancing meaning in life could be an effective intervention for preventing suicide. Ryu and Suh explore the contribution of meaning in life to the relationship between self-disclosure and post-traumatic growth. Specifically, self-disclosure was found to positively influence post-traumatic growth by increasing deliberate rumination, eliciting positive social responses, and enhancing one's sense of meaning in life. This suggests that processing traumatic experiences through meaningful social interactions can lead to personal growth. The importance of intentional reflection and awareness to the experience of meaning in life is also highlighted in processes that promote wellbeing, such as mindfulness. In their article, Li et al. propose a mindfulness-to-meaning theory, which suggests that mindfulness promotes the experience of meaning in life, which, in turn, enhances life satisfaction. This underscores the potential benefits of mindfulness practices in promoting wellbeing and personal growth in everyday life. Osin et al. explore the relationship between ego development, which refers to an individual's capacity to engage with increasingly complex ideas, and eudaimonia, which refers to a sense of wellbeing and flourishing. Their findings suggest that people with higher ego development are better able to make meaning out of their experiences and find purpose in their lives. Lui et al. add another dimension of meaning in everyday life and experience by exploring potential mechanisms that link future orientation and prosociality among youth during the pandemic. Their study suggests that the awareness of meaning and quest for meaning mediate the relationship between future orientation and prosocial behavior. Further extending the importance of social factors in promoting wellbeing, particularly in the context of rapidly changing social structures, Li and He found that social fairness and trust mediated the relationship between social security satisfaction and subjective wellbeing.

## New perspectives and directions in the study of meaning in life

Existing research suggests that individuals can derive meaning from diverse sources, based on their breadth (number of sources) and depth (level of commitment) related to different life domains (Schnell, 2021). Further exploring the importance of varied sources of meaning in life, Karwetzky et al. found that different sources of meaning, such as family, work, or religion, were associated with distinct patterns of brain activity across four age groups. This suggests that the neural mechanisms underlying the experience of meaning may differ depending on the source of that meaning.

The recent COVID-19 pandemic (among other things) has laid bare how meaningful life is grounded in everyday life and activities, be it starting a new hobby, caring for one's community, Zooming with friends and family around the world, or pivoting to a new job so that daily work can feel more purposeful. In parallel, there has been a growing interest among scholars and practitioners alike in how the search for meaning plays out in everyday life and how this approach to studying meaning may shed novel light on the complex puzzle that is meaning. Along these lines, Iso-Ahola and Baumeister delineate leisure as an unrecognized source of meaning in life. By exploring how leisure activities satisfy the basic needs of meaning (i.e., purpose, value, efficacy, and self-worth), the authors structure a foundation for further theory development and empirical testing of various conceptual dimensions which characterize leisure activities.

Religion is considered a central source of meaning for many, offering a structured and comprehensive perspective on life, along with a system of principles, morals, and directives to lead a meaningful and worthwhile existence (Spilka et al., 2003; Krok, 2014). Soenke et al. investigate the role of normative support in atheists' perception of meaning after being reminded of death. It highlights the importance of social support in promoting a sense of meaning in individuals who do not hold religious beliefs. Another perspective on the nature and origins of one's sense of meaning in life is offered by Landau who invites the readers into a philosophical exploration of potential conflicts that may arise when individuals prioritize meaning in life over other values, such as autonomy or personal growth. The article raises ethical considerations and prompts further examination of the concept of meaningfulness.

Finally, two studies suggest new methods for exploring how individuals derive meaning in their everyday lives. Cantarero et al. propose a brief tool to measure an individual's motivation for making sense of their experiences. The authors also suggest that the new scale could be used in a variety of research and clinical settings to better understand the role of meaning motivation in individuals' lives. Finally, Kreiss and Schnell examine the relationship between daily activities and a sense of meaning, suggesting that engaging in meaningful activities is positively associated with wellbeing. Their study is unique, employing an experience-sampling method to collect data on daily activities and their perceived meaning.

Overall, this Research Topic brings together work from multiple disciplines that can answer big-picture questions about the nature and experience of a meaningful life through the study of meaning in everyday life.

## Author contributions

PR-N drafted the manuscript. JH offered revisions and provided approval for the publication of the content. All authors contributed to the article and approved the submitted version.
